# African origin haplotype protective for AD in *APOEe4* carriers: exploring potential mechanisms

**DOI:** 10.1002/alz.091487

**Published:** 2025-01-03

**Authors:** Luciana Bertholim Nasciben, Karen Nuytemans, Marina Lipkin Vasquez, Farid Rajabli, Juan I Young, Derek M. Dykxhoorn, Liyong Wang, William K. Scott, David A. Davis, Regina T Vontell, Margaret A Pericak‐Vance, Anthony J. Griswold, Jeffery M. Vance

**Affiliations:** ^1^ John P. Hussman Institute for Human Genomics, Miller School of Medicine, Miami, FL USA; ^2^ John P. Hussman Institute for Human Genomics, University of Miami Miller School of Medicine, Miami, FL USA; ^3^ Dr. John T. Macdonald Foundation Department of Human Genetics, University of Miami Miller School of Medicine, Miami, FL USA; ^4^ University of Miami Miller School of Medicine, Miami, FL USA; ^5^ 1501 NW 10th Avenue, Miami, FL USA; ^6^ John P. Hussman Institute for Human Genomics, University of Miami Miller School of Medicine, Miami, FL, USA, Miami, FL USA; ^7^ John P. Hussman Institute for Human Genomics, Miami, FL USA

## Abstract

**Background:**

*The* effect size of APOE4 varies across genetic ancestries with African (AFR) local ancestry conferring a lower risk when compared to other ancestries. Recently, we identified a strong effect of the A allele of rs10423769 (with a minor allele frequency of 0.12 in AFR and 0.003 in Europeans), which lowers the risk of AD by 71% in *APOE4* carriers. rs10423769 is 2 MB from *APOEe4* in a region of segmental duplications (SD) containing a cluster of pregnancy specific beta‐1 glycoprotein genes (*PSGs)* and a lncRNA. To gain insights into the mechanism of protection, we characterized the haplotype harboring the variant and investigated differences in methylation and structural variation (SV) between haplotypes.

**Method:**

We used the Alzheimer’s Disease Sequencing Project (ADSP) short read sequencing database to fine map and extend the protective haplotype using Haploview 4.2. In addition, long read sequencing (LRS) with the Oxford Nanopore PromethION was performed on 16 samples of individuals carrying one or two copies of rs10423769_A. LRS was used for SV calling with Sniffles2 and differential methylation analysis using Nanomethphase.

**Result:**

The minimum shared haplotype harboring rs10423769_A allele was ∼21kbp (chr19:43099521‐ 43120243). Though the region is rich in SDs, high quality LRS reads mapped uniquely to the region around the A allele confirming that the haplotype is unique and not duplicated elsewhere in the genome of the sequenced individuals. Moreover, the rs10423769_A haplotype harbors 23 differential CpG sites (fdr<0.05) compared to rs10423769_G, although no differential methylated region was identified. LRS revealed a 140bp insertion of repetitive MEF2 binding motifs. Initial examination demonstrated that the insertion was present in 80% of the A allele carriers.

**Conclusion:**

We have identified an AF‐specific haplotype protective against AD risk conferred by *APOE* ε4. Considering the distance from *APOE* and the complexity of the highly SD region where the variant is located, we have identified the minimum shared haplotype from AFR origin and are investigating the potential effects of SVs and differential CpG sites. Ongoing analyses include 3D chromatin structure and LRS at higher coverage to assemble and resolve the complex genomic context of the allele.

